# Characterization of the bacterial gut microbiota of piglets suffering from new neonatal porcine diarrhoea

**DOI:** 10.1186/s12917-015-0419-4

**Published:** 2015-06-23

**Authors:** Marie Louise Hermann-Bank, Kerstin Skovgaard, Anders Stockmarr, Mikael Lenz Strube, Niels Larsen, Hanne Kongsted, Hans-Christian Ingerslev, Lars Mølbak, Mette Boye

**Affiliations:** National Veterinary Institute, Technical University of Denmark, Bülowsvej 27, 1870 Frederiksberg C, DK Denmark; Department of Applied Mathematics and Computer Science, Technical University of Denmark, Matematiktorvet, Building 324, 2800 Lyngby, DK Denmark; Danish Genome Institute, Skt. Lucas Kirkeplads 8, 8000 Århus, DK Denmark; Danish Pig Research Centre, Danish Agriculture and Food Council, Vinkelvej 13, 8620 Kjellerup, DK Denmark; Present address: Chr. Hansen, Bøge Allé 10-12, 2970 Hørsholm, DK Denmark

**Keywords:** NNPD, Neonatal, Piglet, Diarrhoea, qPCR, Microbiota, Gut Microbiotassay, 454 sequencing

## Abstract

**Background:**

In recent years, new neonatal porcine diarrhoea (NNPD) of unknown aetiology has emerged in Denmark. NNPD affects piglets during the first week of life and results in impaired welfare, decreased weight gain, and in the worst-case scenario death. Commonly used preventative interventions such as vaccination or treatment with antibiotics, have a limited effect on NNPD. Previous studies have investigated the clinical manifestations, histopathology, and to some extent, microbiological findings; however, these studies were either inconclusive or suggested that Enterococci, possibly in interaction with *Escherichia coli*, contribute to the aetiology of NNPD. This study examined ileal and colonic luminal contents of 50 control piglets and 52 NNPD piglets by means of the qPCR-based Gut Microbiotassay and 16 samples by 454 sequencing to study the composition of the bacterial gut microbiota in relation to NNPD.

**Results:**

NNPD was associated with a diminished quantity of bacteria from the phyla Actinobacteria and Firmicutes while genus *Enterococcus* was more than 24 times more abundant in diarrhoeic piglets. The number of bacteria from the phylum Fusobacteria was also doubled in piglets suffering from diarrhoea. With increasing age, the gut microbiota of NNPD affected piglet and control piglets became more diverse. Independent of diarrhoeic status, piglets from first parity sows (gilts) possessed significantly more bacteria from family Enterobacteriaceae and species *E. coli*, and fewer bacteria from phylum Firmicutes. Piglets born to gilts had 25 times higher odds of having NNPD compared with piglets born to multiparous sows. Finally, the co-occurrence of genus *Enterococcus* and species *E. coli* contributed to the risk of having NNPD.

**Conclusion:**

The results of this study support previous findings that points towards genus *Enterococcus* and species *E. coli* to be involved in the pathogenesis of NNPD. Moreover, the results indicate that NNPD is associated with a disturbed bacterial composition and larger variation between the diarrhoeic piglets.

**Electronic supplementary material:**

The online version of this article (doi:10.1186/s12917-015-0419-4) contains supplementary material, which is available to authorized users.

## Background

Neonatal piglet diarrhoea is of significant importance for the pig industry because it causes economic losses due to increased morbidity and mortality, decreased weight gain, and the need for extra medications [1,2]. Obviously, it impairs the welfare of the animals in the short term, but it may also affect their health in the longer term as a consequence of disrupting the normal bacterial succession in the gastrointestinal tract [3].

Bacterial colonization of the mammalian gastrointestinal tract begins at birth [4]. This colonization is a dynamic event, and the bacterial succession is influenced by a number of factors including: mode of delivery, surrounding environment, gestational age, and genetics [5,6]. The colonization of the gut has a major impact on the host’s health and disease; for example, the microbiota helps in the maturation of the gastrointestinal tract and immune system, protects against pathogen colonization through competitive exclusion, and converts otherwise indigestible substances into digestible components that benefit the host [3,5,7,8]. In accordance with these roles, studies of germ-free animals, deficient of normally developed gut microbiota, show animals with an impaired and immature intestinal immune system, in addition to changes in intestinal morphology [8-10].

During the last ten years, increasing attention has been focused on a new neonatal porcine diarrhoea [11-13]. In Denmark, this diarrhoea has been named ‘New Neonatal Porcine Diarrhoea’ (NNPD). What distinguishes NNPD from other types of neonatal piglet diarrhoea are the following: 1) The aetiology is unknown, however, routine diagnostic protocols show that it is not caused by known enteric pathogens such as hemolytic *Escherichia coli*, *Clostridium difficile, Clostridium perfringens* type A or C, coronavirus, rotavirus species A or C, *Cryptosporidium* spp*.*, *Cystoisospora suis*, *Giardia* spp*.*, or *Strongyloides ransomi.* 2) Typical strategies, such as vaccination against enterotoxigenic *E. coli* and *Cl. perfringens* type C or treatment with antibiotics, do not seem to have a noteworthy effect on the diarrhoea. 3) No obvious connection between NNPD and pig farm health status or management has been demonstrated ([14,15] Larsen LE, Nielsen JP, unpublished results).

It is difficult to estimate how widespread NNPD is, mainly because of the unknown aetiology combined with a fluctuating clinical presentation [2], as well as differences in routine laboratory testing [13,15], but also because of the limited number of studies focusing on this issue. Nonetheless, a diarrhoea of much resemblance to NNPD has been described in Sweden and France [13,16,17].

This study is part of an interdisciplinary project investigating the aetiology of NNPD. Kongsted *et al.* suggested the following case-definition of NNPD: *“Non-hemorrhagic diarrhoea during the first week of life, without detection of known infectious pathogens, characterized by milk-filled stomachs and flaccid intestines at necropsy”* [15]. The same author found dissimilarities in the course and severity of NNPD among four pig farms and estimated that affected piglets had a negative average daily weight gain with an increased risk of dying, though this risk was not significant [2]. Jonach *et al.* examined the role of four enteric bacterial pathogens by fluorescence in situ hybridization (FISH) and found that simultaneous colonization of the intestinal mucosa with non-enterotoxigenic *E. coli* (non-ETEC) and *Enterococcus* spp. could be involved in the pathogenesis of NNPD [18]. Finally, several different viral assays tested negative on samples from some of the same NNPD animals that were examined in the aforementioned studies, indicating that common known viruses likely do not contribute to NNPD (Larsen LE, Nielsen JP, unpublished results).

This study investigates whether NNPD is associated with the composition of the gut microbiota obtained from piglets with and without diarrhoea. This was examined by evaluating the overall bacterial composition and relative quantitative distribution of ileal and colonic intestinal content using the Gut Microbiotassay: an assembly of 24 primer sets targeting ribosomal RNA (rRNA) genes (16S and 23S), verified to function with the high-throughput quantitative real-time PCR (qPCR) chip Access Array™ Integrated Fluidic Circuit (AA48.48) from Fluidigm [19]. As the name implies, the assay is designed to target major bacteria phyla and selected taxonomic sub-groups of the gut microbiota. The Gut Microbiotassay provides a quick overview of the distribution, as well as the relative quantity of the gut microbiota in a large number of samples simultaneously. Subsequently, PCR amplicons from ileum and colon of four case piglets and four control piglets were sequenced using 454-technology to acquire deeper taxonomic information. This approach revealed diverse gut microbial profiles associated with piglet diarrhoeic status.

## Methods

### Animals and sample collection

Danish pig farms affected by NNPD were identified from conversations with veterinarians and farm managers. Four pig farms that fulfilled the inclusion criteria listed in were included in the study (for more information on the selection of pigs and herds included in this study, see Kongsted *et al.* [15]). On each pig farm, approximately 15 randomly chosen sows from one farrowing batch (66 in total) were followed for a seven day period following farrowing. All newborn piglets were weighed at the beginning of the trial (average weight 1394 g, SD ± 335 g), and piglets weighing less than 800 g were excluded. All animals were subject to a daily clinical examination that paid special attention to fecal appearance on rectal swabs. Diarrhoea was defined as loose or watery feces. Based on these observations, piglets were characterized as either cases or controls: A case piglet had suffered from diarrhoea for at least two consecutive days, whereas a control piglet had never experienced diarrhoea. The inclusion criterion applied to case piglets resulted in a greater number of case piglets from gilts (approximately 66%), as piglets born to multiparous sows did not suffer from diarrhoea to the same extent as piglets born to gilts. Control piglets were as far as possible collected from litters without diarrhoea. In total 50 control piglets and 52 case piglets were selected (Table 1) and brought to the Danish Pig Research Centre, Kjellerup, for euthanization and necropsy. For ethical reasons, farmers were allowed to treat piglets for any disorder if necessary, on the condition that any such treatments were recorded for individual animals. During necropsy, intestinal contents were collected from the distal small intestine (ileum) and the large intestine (colon) of each animal and stored at −80°C until further analysis.

**Table 1:** **Piglets included in the study**

**Age in days**	**3**		**4**		**5**		**6**		**7**		**Total**			
											**Gilts**		**Sows**	
**Diarrhoea**	**−**	**+**	**−**	**+**	**−**	**+**	**−**	**+**	**−**	**+**	**-**	**+**	**-**	**+**
**Pig farm 1**	6	7			7	6					**0**	**4**	**13**	**9**
**Pig farm 2**			2	2	6	6			4	4	**2**	**12**	**10**	**0**
**Pig farm 3**			7	8			6	6			**0**	**10**	**13**	**4**
**Pig farm 4**	8	8			4	5					**4**	**8**	**8**	**5**
**Total**	**14**	**15**	**9**	**10**	**17**	**17**	**6**	**6**	**4**	**4**	**6**	**34**	**44**	**18**

The number of piglets is listed for each pig farm in accordance with age in days: (−) Control piglets without diarrhoea, (+) Case piglets with diarrhoea for ≥2 days. The column “Total” summarizes the number of piglets born to gilts or sows (multiparous sows) by farm.

**Pig farm inclusion criteria for studying NNPD[**15**]:**

- A stock of more than 400 sows
- Routinely vaccinate^1^ against ETEC^2^ and *Cl. perfringens* type C
- Farrowing units proven to be PRRS^3^ virus negative as demonstrated in blood samples tested by ELISA^4^/IPT^5^ or PCR.
- NNPD has been a problem for a period of minimum 3–6 months
- Management-related diarrhoea has been excluded
- Traditional interventions such as vaccines and antibiotics have limited effect on the diarrhoea
- At least 30% of the litters are affected
- The piglets suffer from diarrhoea during the first week of life
- Random check of five case piglets should test negative for ETEC, *Cl. perfringens* type C and Rotavirus by routine diagnostic examination

### DNA extraction

Based on development work comparing various extraction methods, the Maxwell® 16 LEV Blood DNA Kit (Promega, Madison, WI, USA) was determined to be the optimal method, based on providing sufficient DNA yields and acceptable purities. According to manufacturer’s recommendations, total DNA was extracted from intestinal content using this kit. A total of 200 mg of intestinal contents were suspended in 600 μl PBS and vortexed until visually homogeneous. The samples were centrifuged for 2 min at 200 × *g*, and the supernatant was transferred to new tubes. A volume of 350 μl of lysis buffer was then added, and the bacterial cells were lysed by bead beading (Tissuelyser II, Qiagen, Hilden, Germany) for 2.5 min at 20.0 hertz with a 5 mm steel bead (Qiagen). Subsequently, samples were centrifuged for 1 min at 1000 × *g* at 4°C, after which the supernatant was transferred to novel 2 ml tubes and mixed manually with 30 μl Proteinase K (Promega). Next, samples were incubated for 30 min at 56°C, and centrifuged at 13 000 × *g* for 1 min. The entire suspension was transferred to the sample inlets on the cartridge, and 50 μl of elution buffer was added to the bottom of the elution tubes. The cartridges were prepared according to the manufacturer’s instructions [20] and the settings “Research Mode”, “LEV Mode”, “DNA”, and “Blood/Cell” were selected for DNA extraction using the Maxwell® 16 Instrument (Promega). Finally, tubes were centrifuged at 20 000 × *g* for 3 min to settle any magnetic bead leftovers, and the DNA was moved to new tubes. DNA concentration and purity was measured using the Nanodrop® ND-1000 (NanoDrop Technologies Inc., Wilmington, Germany) spectrophotometer, and DNA was stored at −20°C until further analysis.

### Analysis of bacterial profiles with use of the Gut Microbiotassay

To obtain a bacterial profile of the intestinal content, this study used the Gut Microbiotassay designed for the AA48.48 [19] with a few modifications. In order to improve the performance of the primer sets “Domain Bacteria B V4-V5” and “Phylum Firmicutes” degenerate nucleotides were introduced: Nucleotide 11 was changed from C to M (C/A), and nucleotide 5 from T to Y (T/C), (5′-3′ direction), in the forward or reverse primer, respectively (Additional file 1). All remaining primer sets were identical to those previously published as ‘The Gut Microbiotassay’ [19]. Primers were purchased from Eurofins MWG Synthesis GmbH (Ebersberg, Germany) and stored at −20°C.

In brief, the AA48.48 is a qPCR platform that is capable of running 48 × 48 = 2304 individual reactions simultaneously, and it enables quick and easy library preparation for 454 sequencing [21]. The AA48.48 was processed and prepared following the ‘Access Array System™ User Guide’ [21], with and without adding the Access Array Barcode Library for the 454 GS FLX Titanium Sequencer (454BL), as previously described [19]. Primers targeting bacteria at the taxonomic level of species were not tagged or included for sequencing, as these amplicons were regarded as contributing little information due to their specificity. However, analysis at the species-level was possible using the information obtained at higher taxonomic levels. All samples were diluted to 50 ng/μl with nuclease-free water (Ambion Inc., Austin, USA). Primers were diluted to 4 μM with 20× Access Array Loading buffer and nuclease-free water (Ambion Inc.). Master mix was a mixture of: 10× FastStart High Fidelity Reaction Buffer with 18 mM MgCl_2_ (Roche Diagnostics, GmbH, Mannheim, Germany), 25 mM MgCl_2_ (Roche), DMSO (Roche), 20× Access Array Loading Reagent (Fluidigm Corporation, South San Francisco, CA, USA), 50× ROX (Invitrogen Corporation, Carlsbad, CA, USA), 20× EvaGreen® (Biotium, Inc., Hayward, CA, USA), 10 mM PCR Grade Nucleotide Mix (Roche), and 5 U/μl FastStart High Fidelity Enzyme Blend (Roche), in final concentrations of 1×, 2.7 mM, 5%, 1×, 0.5×, 1×, 200 μM, and 0.05 U/μl, respectively. Sample mix was prepared from 3 μl master mix, 1 μl 454BL (2 μM) (Fluidigm), and 1 μl DNA (50 ng/μl) as the very last step before running the array (without the 454BL: 4 μl master mix and 1 μl DNA (50 μg/μl)). When the PCR reaction had finished, the barcoded PCR amplicons were harvested and stored at −20°C.

### Next generation sequencing

Samples from two representative animals were selected from each farm: one piglet with NNPD and one piglet without NNPD, giving a total of 16 samples to be sequenced (when samples from both the ileum and colon of each animal were included). Piglets were chosen to be approximately the same age (5 or 6 days old). Concentrations of the respective PCR amplicons for each animal were determined using the Agilent 1000 chip (Agilent Technologies, Waldbronn, Germany). Amplicons were subsequently pooled in equal concentrations and size-separated by running the amplicons for 86 min, 90 V, in a 0.7% Seakem® LE Agarose gel (Lonza Rockland, Rockland, ME, USA) followed by incubation for 30 min in 0.0004% ethidium bromide for staining. By means of UV radiation from the Bio-Rad Universal hood II (Segrate, Milan, Italy) gel bands were visualized and bands spanning the size range of the primer products (200–900 base pairs) were excised. Finally, the Qiaquick Gel Extraction Kit (Qiagen) was used to purify DNA from the gel. This pool of 1615.7 ng DNA (260/280 nm-ratio: 1.97) derived from ileal and colonic luminal contents of 8 different animals was sequenced on a half PicoTiterPlate™ by a 454 GS FLX Titanium Sequencer (Roche) via LGC Genomics (GmbH, Berlin, Germany).

## Data analysis

### Relative quantification of quantification cycle (Cq) values

Data analysis was conducted as described in a previously published methodology article [19]: Raw Cq values were exported from ‘Fluidigm Real-Time PCR Analysis’ software version 3.0.2 to Microsoft Excel. To even out possible variation between the AA48.48 runs, all Cq values were normalized to an Interplate Calibrator. Next, Cq values from each sample were normalized to their respective “Domain Bacteria B V4-V5” primer set, thereby calculating the relative quantification.

### Principal Component Analysis on normalized Cq data

Principal Component Analysis (PCA) was conducted in the software package R, version 3.1.0 [22]. All normalized data were initially transformed with the natural logarithm (ln). Next, primer sets with less than 50% recorded Cq values were excluded from the analysis, and in the same manner, samples that resulted in less than 50% registered Cq values were removed. For the remaining primer sets, missing values were substituted with the lowest registered primer-specific value. Data were scaled by the individual primer mean and standard deviation for PCA. The number of components to be investigated further was determined by visual inspection of scree plots of eigenvalues; based on an inflection point or when the components explained > 60% variance (Additional file 2). All possible combinations of included principal components were visualized to ensure that no important clustering was overlooked. The number of components used for graphical representation in the PCA was set at two to ease interpretation. Samples used for 454 sequencing were pinpointed from the PCA results. In order for samples to be considered for sequencing the samples were represented with luminal content from both ileum and colon, and none of these were allowed to have outlying coordinates in the PCA, but were somewhat in the centre of its respective category (Control_colon; Case_colon; Control_ileum; and Case_ileum). Linear discriminant analysis (LDA) based on leave-one-out cross-validation was used to classify observations into cases or controls by their Fluidigm-derived microbial profile and to estimate how well this classification corresponded with the actual status of the sample. This classification was followed up with a multivariate analysis of variance (MANOVA) using Wilks Lambda statistic.

### Statistics on the Gut Microbiotassay

Bacterial profiles obtained from the Gut Microbiotassay were used to study the gut microbiota of piglets with and without NNPD. For the statistical calculations all values were initially ln-transformed. A linear mixed-effect model was then applied to each primer set separately with randomized effect of herd origin:

1 $$ \ln \left({Y}_i\right)={a}_{Gut\  Section(i), Status(i)}+{a}_{Gilt(i)}+{a}_{Treatment(i)}+{\beta}_{Status(i)}\cdot Ag{e}_i+\beta \cdot diarrhoe{a}_i+\eta \cdot {Y}_{herd(i)}+{\varepsilon}_i,i=1,\dots, 102 $$,

where *Status*(*i*) indicates the status of the *i*’th animal, and where *Y*_*herd*(*i*)_ is a normalized Gaussian stochastic variable indicating the randomized effect of herd, indexed by the herd of the *i*’th animal, so that animals from the same herd share the random effect. The model is applied for different responses *Y*, as listed in the first column in Table 2. The following variables were included as deterministic effects in the model: Gut section (ileum versus colon), Status (diarrhoeic versus healthy), Gilt (born to a gilt versus born to a multiparous sow), Percentage of diarrhoea (number of days with diarrhoea/days of age × 100), Treatment (treated versus non-treated animal – this category only involves case piglets), Age (days). The interaction between Gut section and Status was also included to examine if possible diarrhoea-causing agents could be traced to a specific gut section. Additionally, the interaction between Status and Age was included to study whether the effect of age differed between case and control piglets. For each primer set as response variable, the model was reduced by backwards stepwise elimination, resulting in a series of primer-specific final models. Model reduction was performed as likelihood ratio tests. Model fit for each primer set was assessed through residual analysis of the model residuals corrected for random effects and the estimated random effects themselves, using standard techniques. These analyses were consistent with standard model behavior. The effect of gender and birth weight in relation to status was also evaluated. Significances in the final models were reported through *p*-values.

**Table 2:** **Significant findings from the Gut Microbiotassay and 454 sequencing results displaying similar trends**

**Primer set and respective 454 sequencing findings**	**Gut section estimate (ileum vs. colon)**	**Gut section** ***p-*** **value**	**Status estimate (diarrhoeic vs. healthy)**	**Status** ***p*** **-value**	**Gilt estimate (gilt vs. multiparous sow)**	**Gilt** ***p*** **-value**	**Percentage of diarrhoea estimate** ^**#**^	**Percentage of diarrhoea** ***p*** **-value**	**Age estimate**	**Age** ***p*** **-value**	**Antibiotics estimate (treated vs. untreated)**	**Antibiotics** ***p*** **-value**
**Domain Bacteria B** ^**268**^	0.12 [0.08,0.17]	<0.0001		NS		NS		NS	1.21 [1.01,1.44]	0.04		NS
**Phylum Firmicutes** ^**183**^	2.37 [1.63,3.33]	<0.0001	0.58 [0.36,0.89]	0.02	0.55 [0.33,0.87]	0.01		NS	1.34 [1.13,1.59]	0.001		NS
**Class Bacilli** ^**125**^	3.31 [1.98,5.20]	<0.0001	0.29 [0.18,0.46]	<0.0001		NS		NS	1.62 [1.27,2.03]	0.0001		NS
**Genus** ***Enterococcus*** ^**27**^	3.51 [1.25,7.91]	0.02	24.15 [8.50,54.73]	<0.0001		NS		NS		NS		NS
species *E. hirae* (3.36/11.11)												
species *E. faecium* (9.87/129.60)												
species *E. durans* (15.57/255.00)												
species *E. mundtii* (113.00/179.00)												
**Genus** ***Streptococcus*** ^**47**^	2.49 [1.53,3.82]	0.0002	0.09 [0.02,0.27]	0.0002	0.55 [0.29,0.93]	0.03	1.03 [1.01,1.05]	0.008	0.75 [0.62,0.90]	0.003		NS
species *S. gallolyticus subsp. gallolyticus* (0.29/0.22)												
species *S. alactolyticus* (0.27/0.18)												
species *S. uberis* (0.68/0.33)												
**Genus** ***Lactobacillus*** ^**79**^	4.97 [2.91,7.94]	<0.0001	0.29 [0.17,0.46]	<0.0001		NS		NS	1.75 [1.36,2.20]	<0.0001		NS
species *L. acidophilus* (0.35/0.06)												
**Family Clostridium cluster I** ^**17**^	3.32 [1.37,6.79]	-	0.36 [0.04,1.39]	-		NS	1.03 [1.00,1.05]	0.04		NS		NS
species *Cl. perfringens;* ATCC 13124 (1.18/0.47)												
**Species** ***Cl. perfringens***	9.31 [3.40,20.66]	-	1.41 [0.51,3.16]	-		NS		NS	0.67 [0.49,0.90]	0.009		NS
**Family Clostridium cluster IV** ^**64**^	0.12 [0.05,0.25]	<0.0001	0.13 [0.06,0.25]	<0.0001		NS		NS	1.41 [1.02,1.91]	0.04		NS
species *Ruminococcus bromii* (0.00/0.26)												
**Family Clostridium cluster XIV** ^**81**^	0.08 [0.05,0.14]	<0.0001		NS		NS	0.99 [0.98,0.99]	0.001		NS		NS
species *Roseburia inulinivorans* (1.59/0.02)												
**Phylum Bacteroidetes** ^**126**^	0.02 [0.01,0.05]	-	0.12 [0.05,0.26]	-		NS		NS		NS		NS
**Genus** ***Bacteroides*** ^**65**^	0.03 [0.01,0.07]	-	0.20 [0.08,0.43]	-		NS		NS		NS		NS
species *Bacteroides pyogenes* (0.02/0.33)^†^												
species *Bacteroides rodentium* (0.46/0.00)^†^												
species *Bacteroides xylanisolvens* (0.94/0.43)^†^												
species *Porphyromonadaceae bacterium* C941 (0.00/0.01)^†^												
**Phylum Actinobacteria** ^**74**^	2.84 [1.64,4.60]	<0.0001	0.29 [0.17,0.46]	<0.0001		NS		NS	0.80 [0.65,0.99]	0.04		NS
species *Bifidobacterium boum* (0.41/0.06)												
species *Corynebacterium kutscheri* (0.39/0.34)												
**Family Bifidobacteriaceae** ^**12**^	2.56 [1.28,4.61]	0.007		NS		NS		NS		NS		NS
**Phylum Fusobacteria** ^**16**^	0.20 [0.10,0.34]	<0.0001	2.24 [1.11,4.05]	0.02		NS		NS	0.69 [0.53,0.88]	0.004	0.20 [0.05,0.52]	0.002
species *F. mortiferum* (2.25/1.21)												
**Class Beta- and Gammaproteobacteria** ^**109**^	2.54 [1.63,3.79]	<0.0001	0.72 [0.12,2.55]	-		NS		NS	0.69 [0.52,0.88]	-		NS
**Family Enterobacteriaceae** ^**89**^	4.03 [2.50,6.16]	<0.0001	0.27 [0.04,1.02]	-	2.12 [1.09,3.75]	0.03		NS	0.54 [0.40,0.71]	-		NS
species *Escherichia coli* (8.61/6.22)												
species *E.coli;* HQ219945.1.1457 (11.46/5.00)												
species *E. coli* DEC8A (6.22/5.16)												
**Species** ***E. coli***	3.56 [2.05,5.76]	<0.0001	0.29 [0.02,1.26]	-	2.96 [1.39,5.56]	0.004		NS	0.58 [0.41,0.80]	-		NS
**Class Deltaproteobacteria** ^**1**^		NS		NS		NS	0.98 [0.97,1.00]	0.04		NS	0.08 [0.00,0.48]	0.02
species *Desulfovibrio piger* (0.00/0.00)^†^												

Estimated fold changes as the estimates of the exponentiated effect parameter, corresponding 95% confidence intervals [], and *p-*values. Superscripted numbers equal the total number of OTUs belonging to the respective taxonomic group identified by sequencing amplicons generated by the Gut Microbiotassay**.** OTU-ratio between diarrhoeic and healthy piglets for ileum and colon, respectively, listed in parenthesis. ^†^indicate a significant effect of interaction (Table 3). Species mark with † are not consistent findings. ^#^Estimated fold changes for this category are calculated as: *γ* ^*percentage points*^, where the parameter *y* from column 8 is the estimate of the exponential *eβ* of the estimated effect parameter *β* for diarrhoea in formula (1). The diarrhoeic percentage points range from 0 to 100.

Previous studies have suggested that simultaneous colonization with *E. coli* and Enterococci species may be a cause of neonatal porcine diarrhoea [13,18,23,24]. This co-occurrence was also implied using the aforementioned model (as bacteria belonging to these taxonomical groups displayed the highest estimated fold change differences being more numerous in diarrhoeic piglets compared to control piglets (Tables 2 and 3)), thus, this was further investigated. A logistic regression model with randomized effect of herd origin, fit by penalized quasi-likelihood, was used to determine if the odds of NNPD was associated with the following variables: Gut section, Gilt, Age, genus *Enterococcus*, class Beta- and Gammaproteobacteria, family Enterobacteriaceae, species *E. coli*, as well as possible interaction in between bacteria, and between bacteria and gut section:

**Table 3:** **Estimated fold changes, 95% confidence intervals [], and** ***p-*** **values of significant interactions from the Gut Microbiotassay**

**Primer set**	**Gut section: status estimate (ileum, diarrhoeic)**	**Gut section: status estimate (colon, diarrhoeic)**	**Gut section: status** ***p-*** **value**	**Status: age estimate, day 3**	**Status: age estimate, day 4**	**Status: age estimate, day 5**	**Status: age estimate, day 6**	**Status: age estimate, day 7**	**Status: age estimate, mean age**	**Status: age** ***p*** **-value**
**Family Clostridium cluster I**	0.07 [0.01,0.31]	0.27 [0.04,1.39]	0.01							NS
**Species** ***Cl. perfringens***	0.33 [0.13,0.80]	1.41 [0.51,3.16]	0.04							NS
**Phylum Bacteroidetes**	1.00 [0.39,2.42]	0.12 [0.05,0.26]	0.0009							NS
**Genus** ***Bacteroides***	0.73 [0.13,0.80]	0.20 [0.08,0.43]	0.04							NS
**Class Beta- and Gammaproteobacteria**			NS	3.12	5.63	10.21	18.60	34.37	4.52 [5.05,11.64]	0.0007
**Family Enterobacteriaceae**			NS	1.81	3.80	8.07	17.29	38.07	4.52 [3.19,9.69]	<0.0001
**Species** ***E. coli***			NS	1.98	4.38	9.81	22.42	53.95	4.47 [3.39,11.85]	0.0004

This table is an extension of Table 2, and the estimated fold changes are the estimates of the exponentiated effect parameter.

2 $$ logit\left({p}_i\right)={a}_{Gut\  section(i)}+{a}_{Gilt(i)}+{\beta}_{Age}\cdot Ag{e}_i+{\displaystyle \sum_{j=1}^4{\beta}_{j, Gut\  section(i)} bacteri{a}_{j,i}+{\displaystyle \sum_{k=1}^4{\displaystyle \sum_{\ell \ne k}{\beta}_{k,l} bacteri{a}_{k,i} bacteri{a}_{\ell, i}+\eta {Y}_{herd(i)}}}} $$,

where *bacteria*_1_-*bacteria*_4_ are genus *Enterococcus*, class Beta- and Gammaproteobacteria, family Enterobacteriaceae, and species *E. coli*, respectively, where *bacteria*_*j,i*_ is the value of *bacteria*_*j*_ animal *i*, where *Age*_*i*_ is the age in days of animal *i*, and where *Y*_*herd*(*i*)_ is the random effect component corresponding to the herd of animal *i*.

The models were validated through graphical procedures. A *p*-value of less than 0.05 was considered significant. Statistical analyses were performed in R [22].

### Bioinformatics analysis of 454 sequencing data

Sequencing data available at NCBI Sequence Read Archive [NCBI:SRP044282] were analyzed using the as yet unpublished open source package BION-meta (in preparation, N. Larsen, Danish Genome Institute, Denmark) (Additional file 3). This program facilitates quick and easy bioinformatics analysis, and BION-meta has previously been applied to a similar dataset [19]. However, in addition to matching all sequences against the ribosomal small subunit (SSU) Silva dataset, suited for 16S rRNA gene targeting primer sets, BION-meta was advanced to encompass searches in the ribosomal large subunit (LSU) Silva dataset, thereby accommodating the primer sets targeting the 23S rRNA gene used in the Gut Microbiotassay [25]. The BION-meta workflow included the following elements: 1) separation by sample barcodes and primer tags; 2) removal of primer remnants and bases at the ends with lower than 96% quality (phred value ~14), as well as sequence filtering by length (200) and filtering by minimum base quality of 96%.; 3) removal of chimeric sequences; 4) separation of each sample by matching it with the phylogenetic primer(s); 5) matching all sequences against the SSU and LSU Silva datasets and producing a table with the highest 1% similarities for each query; and 6) mapping the similarities to the Silva SSU and LSU taxonomies, identifying consensus operational taxonomic units (OTUs).

To examine the gut microbiota in further details, BION-meta data were analyzed by Principal Coordinates Analysis (PCoA). This was conducted by applying the vegan package in R using Bray-Curtis distances on the untransformed sequence reads followed by k-means clustering [26].

Due to the hierarchical taxonomic design of the Gut Microbiota, where several primers in taxonomic lineage potentially target the same bacterial organism, a number of primer sets were chosen for this expanded diversity analysis (referred to as “grand data”): phylum: Actinobacteria, Bacteroidetes, Fusobacteria, and Spirochaetes; class: Deltaproteobacteria, and Epsilonproteobacteria; family: Clostridium cluster I, cluster IV, and cluster XIV, and Enterobacteriaceae; genus: *Enterococcus*, *Lactobacillus*, *Streptococcus*. These primer sets were selected because they provide the most comprehensive taxonomic information with the least taxonomic trespassing that would result in distortion and consequentially misinterpretation of the results.

## Results

From four well-managed Danish conventional pig farms a total of 201 ileal and colonic intestinal content samples were collected from 50 control piglets without NNPD and 52 case piglets suffering from NNPD (3 samples were lost between sampling and the laboratory). Of the case piglets, 25 were treated with broad-spectrum antibiotics intramuscularly, depending on their diagnosis and according to herd treatment practices. Piglets were three to seven days old and were all reared with their biological dam and siblings [15].

**Figure 1:**
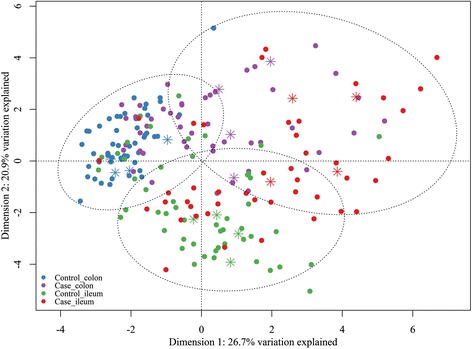
PCA score plot of ileal and colonic content of piglets with (case) and without (control) NNPD. PCA scores are generated from normalized relative values of ileal and colonic content of case and control piglets obtained from the Gut Microbiotassay. Asterisks symbolize samples selected for 454 sequencing. Circles represent K-means clustering. For further information see Additional files 2 and 4.

A bacterial taxonomic profile was obtained from both gut sections of each animal using the Gut Microbiotassay targeting rRNA genes of major bacterial groups in the mammalian intestine. Normalized Cq data were used for PCA. After excluding the primer sets with less than 50% recorded Cq values (“Class Epsilonproteobacteria”, “Phylum Verrucomicrobia”, and “Domain Archaea”), as well as the “Domain Bacteria A” and “Domain Bacteria B” primer sets (which did not contribute any information to the PCA analysis), 19 primer sets remained for the data analysis. In addition, 12 samples were removed from the dataset as a consequence of having too few Cq registrations. From the PCA scores plot (see Figure 1, for further information on the major principal components in the PCA see Additional file 2), four case animals (three of which were treated with antibiotics) and four control animals were randomly chosen to be representatives for their respective herds. Detailed taxonomic information was acquired for these 16 samples using 454 next generation sequencing.

### The Gut Microbiotassay

The significant findings from the Gut Microbiotassay are presented in Table 2 and significant effects of interaction as investigated in formula (1) are presented in Table 3, consequently these two tables are intertwined and should be read together. Table 2 summarizes the estimated fold changes as the estimates of the exponentiated effect parameter, 95% confidence intervals, and *p-*values of these results, and Table 3 lists the same parameters for all significant interactions.

From the relative quantitative results generated by the Gut Microbiotassay, it was evident that luminal content from the large intestine possessed a significantly higher bacterial load than luminal content from the small intestine (expressed by domain Bacteria B, *p* < 0.0001), regardless of diarrhoeic status (Table 2).

NNPD was associated with a diminished quantity of bacteria from the phyla Actinobacteria (*p* < 0.0001) and Firmicutes (*p =* 0.02). Firmicutes comprised the following: class Bacilli (*p* < 0.0001), genus *Lactobacillus* (*p* < 0.0001), genus *Streptococcus* (*p* = 0.0002), and family Cl. cluster IV (*p* < 0.0001). However, genus *Enterococcus* was estimated to be more than 24 times more abundant in diarrhoeic piglets (*p* < 0.0001). The number of bacteria from the phylum Fusobacteria (*p* = 0.02) was also doubled in piglets suffering from diarrhoea (Table 2).

It was investigated whether the effect of diarrhoeic status differed between gut sections through a statistical interaction term (Table 3). In both case and control piglets, there were generally more members from phylum Bacteroidetes and genus *Bacteroides* in the colon compared with the ileum (Table 2). Nonetheless, case piglets had a reduced number of Bacteroidetes and *Bacteroides* compared to control piglets, and the depletion of these bacteria was located to the colon (*p* = 0.0009 and 0.04, respectively), while no significant depletion was detected in the ileum (Table 3). Overall, there were greater numbers of family Cl. cluster I and species *Cl. perfringens* in control piglets compared with case piglets, except for *Cl. perfringens*, which was more numerous in the colon of case piglets, though not significantly. The numbers of Cl. cluster I and *Cl. perfringens* were significantly reduced in the ileum of case piglets compared with control piglets (*p* = 0.01, 0.04) (Table 3). Control piglets generally exhibited a disparity in the number of bacteria present in the ileum versus the colon, while the difference in bacterial abundance between gut sections was more negligible in case piglets. It should be noted that the preceding results all relate to circumstances of ‘all other things being equal’.

Independent of diarrhoeic status, piglets from first parity sows possessed significantly more bacteria from family Enterobacteriaceae (*p* = 0.03) and species *E. coli* (*p* = 0.004), and fewer bacteria from phylum Firmicutes (*p* = 0.01), hereof genus *Streptococcus* (*p* = 0.03) compared to multiparous sows (Table 2).

Bacteria from family Cl. cluster XIV (*p* = 0.001) and class Deltaproteobacteria (*p* = 0.04) were depleted, but bacteria belonging to genus *Streptococcus* (*p* = 0.008), and family Cl. cluster I (*p* = 0.04) were elevated in case piglets that suffered from an increasing percentage of diarrhoea in their lifetime. In Table 2 the estimated fold changes may be calculated as: *γ*^*percentage points*^, where the parameter *y* from column 8 is the estimate of the exponential *e*^*β*^ of the estimated effect parameter *β* for diarrhoea in formula (1). The diarrhoeic percentage points range from 0 to 100.

Diarrhoeic piglets treated with broad-spectrum antibiotics had a reduced presence of Fusobacteria (*p* = 0.002), and Deltaproteobacteria (*p* = 0.02), (Table 2).

Regardless of diarrhoeic status, the quantity of bacteria in the intestine generally increased with increasing age (*p* = 0.04). The older the piglet the more bacteria from the following groups: phylum Firmicutes (*p* = 0.001), class Bacilli (*p* = 0.0001), genus *Lactobacillus* (*p* < 0.0001), and class Cl. cluster IV (*p* = 0.04). Bacteria that diminished from the gut microbiota with increasing age were as follows: genus *Streptococcus* (*p* = 0.003), species *Cl. perfringens* (*p* = 0.009), phylum Actinobacteria (*p* = 0.04), and phylum Fusobacteria (*p* = 0.004) (Table 2).

**Figure 2:**
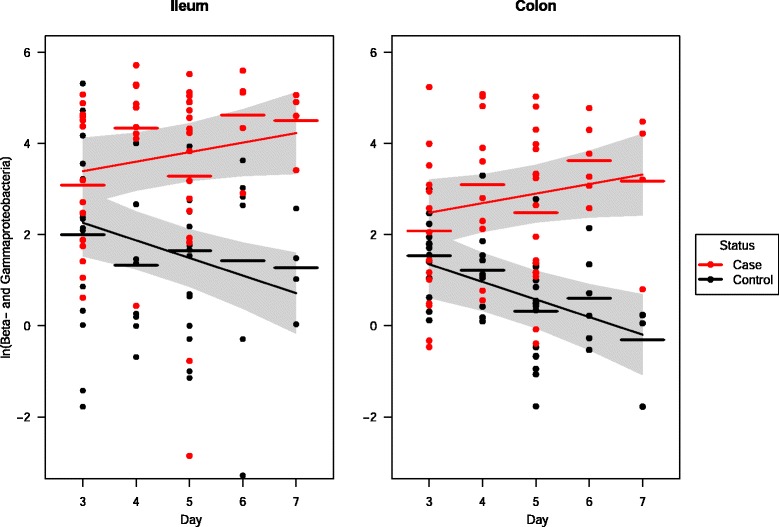
Age-related changes of Beta- and Gammaproteobacteria in luminal content of piglets with and without NNPD. Relative amount of class Beta- and Gammaproteobacteria (transformed with the natural logarithm) in ileal and colonic content of all piglets included in the study, plotted as function of the age of the piglets. Case and controls are piglets with and without diarrhoea, respectively. Age-related model regression lines are depicted with corresponding 95% confidence intervals as shaded areas. Horizontal bars show means for a given age and group.

In addition, the interaction between Status and Age was examined (Table 3). There was a significant difference between control piglets and case piglets over time. As piglets aged, it was estimated that apparently increasing numbers of class Beta- and Gammaproteobacteria (slope: 0.21 log units/day), family Enterobacteriaceae (0.12) and species *Escherichia coli* (0.25) colonized the intestine of case piglets, while the numbers of these bacteria were estimated to decrease for control piglets (*p* = 0.004, *p* < 0.0001, *p =* 0.001, respectively). Figure 2 illustrates the abundance of class Beta- and Gammaproteobacteria in ileal and colonic luminal content in relation to piglet age, segregated according to diarrhoeic status. However, while the differences in age effects between control and case piglets were highly significant (*p* = 0.0007, 0.0001, 0.0004), the three estimated positive slope values for diarrhoeic piglets were not significantly greater than zero (*p* = 0.11, 0.39, 0.12).

The effects of birth weight and gender were investigated, and no significant results were discovered.

Table 4 lists the significant results calculated from the logistic regression model (2) investigating whether NNPD was associated with genus *Enterococcus*, class Beta- and Gammaproteobacteria, family Enterobacteriaceae, species *E. coli*, possible co-occurrence of bacteria, Gut section, Gilt, and Age. The model fitted the data according to standard model control measures. NNPD was found to be significantly associated with the presence of genus *Enterococcus* (*p* = 0.009), and there was also a significant effect of the co-occurrence of this genus and species *E. coli* (*p* = 0.02). Though the interaction apparently had a slightly diminishing effect on the probability of being recognized as an NNPD piglet, it actually contributed to the risk in the majority of cases with increasing values of *E. coli.* This result is explained by the logged values of Enterococci, which were negative in 89% of the data. Finally, there was a significant effect of the interaction between class Beta- and Gammaproteobacteria and the colon, suggesting that NNPD piglets are differentiated from control piglets by colonization of the colon with class Beta- and Gammaproteobacteria (*p* = 0.001). All things being equal, piglets born to gilts had 25 times higher odds of having NNPD compared with piglets born to multiparous sows (*p* < 0.0001).

**Table 4:** **NNPD status associated with selected bacteria and relevant variables**

**ln(variable)**	**Estimate**	**95% confidence interval**	***p-*** **value**
Intercept	−0.98	[−3.10,1.13]	-
Species *E. coli*	−0.11	[−0.42,0.65]	-
Genus *Enterococcus*	0.34	[0.09,0.59]	0.009
*Enterococcus*: *E. coli*	−0.12	[−0.23,-0.02]	0.02
Beta- and Gammaproteobacteria: colon	0.82	[0.33,1.30]	0.001
Gilt	3.23	[1.99,4.46]	<0.0001

Of the following variables tested, genus *Enterococcus*, class Beta- and Gammaproteobacteria, family Enterobacteriaceae, species *Escherichia coli*, Gut section, Gilt, and Age, as well as possible interactions between bacteria, NNPD was found to be significantly related to the variables mentioned below. Numbers are transformed with the natural logarithm (ln). The odds ratio can be calculated as the exponentiated estimate.

### PCA

**Figure 3:**
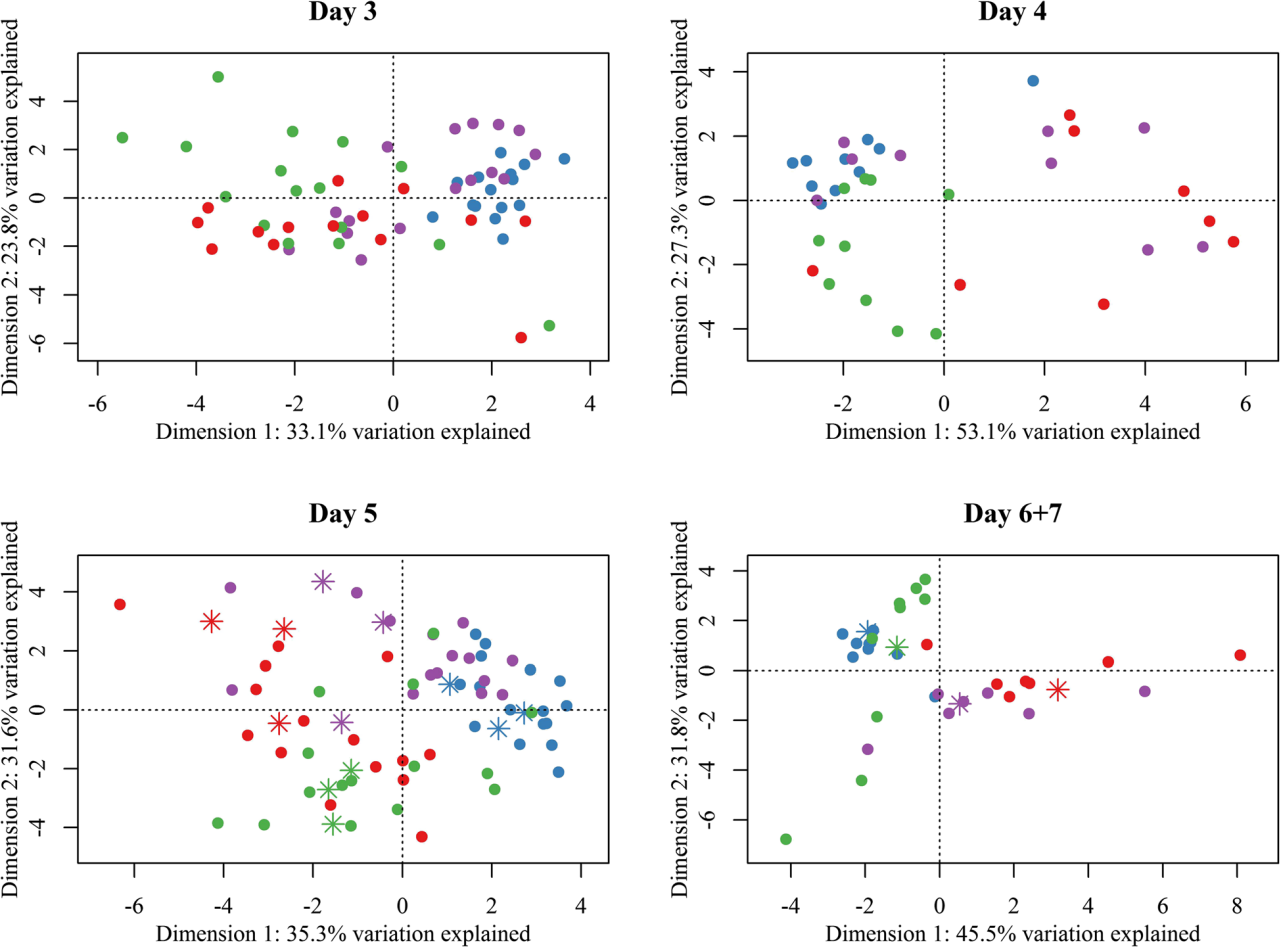
PCA score plot of the gut microbiota segregated on gut section according to age in days. PCA scores from normalized relative values of ileal and colonic content of case and control piglets in relation to age in days obtained from the Gut Microbiotassay. Blue: Control colon, Purple: Case colon, Green: Control ileum, Red: Case ileum. Asterisks symbolize samples selected for 454 sequencing. Samples cluster primarily according to diarrhoeic status with increasing age. For further information see Additional files 2 and 4.

Additional file 2 summarizes the loadings, the eigenvalues, and the variance explained by the major principal components for all PCA analyses. Principal Component Analysis of data from the 19 primer sets showed no clustering in relation to pig farms (results not shown). The major loading scores in the PCA were explained by the primer sets: component 1: phylum Bacteroidetes (Control) and family Enterobacteriaceae (Case), component 2: genus *Lactobacillus* (Ileum), and genus *Bacteroides* (Colon)*.* Generally, the control large intestine was characterized by a gut microbiota that clustered together, whereas the bacterial composition of the small intestine from control piglets was more diverse (Figure 1). Diarrhoeic gut sections were more scattered compared to non-diarrhoeic ones in the PCA plot, demonstrating that NNPD seems to be associated with a disturbed bacterial composition and larger variation between the diarrhoeic piglets. Additional file 4 lists LDA results. According to LDA, 70% and 83% of samples were correctly classified as cases and controls, respectively, in Figure 1 (*p* < 0.0001). When looking at the microbial composition in piglets at different ages (three to seven days old), separation became increasingly more apparent with age, in relation to diarrhoeic status as the predictive value calculated from LDA improved with age (Figure 3, Additional file 4).

### Next generation sequencing

BION-meta processing including de-multiplexing, cleaning, and chimera checking resulted in 279 103 reads for mapping (Additional file 3).

OTUs displaying similar trends to data obtained from the Gut Microbiotassay are included in Table 2. To be represented the OTUs have to be a somewhat consistent finding in piglets, irrespective of the variables in relation to diarrhoea, and the reads should be represented in a noteworthy number.

**Figure 4:**
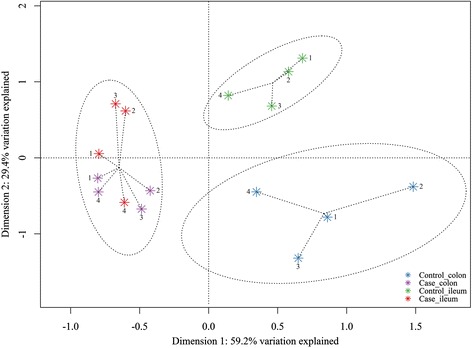
PCoA score plot showing similarity of composition of the gut microbiota from sequenced samples. Grand data of ileal and colonic luminal content of a case piglet and a control piglet from each herd included in the study. Numbers refer to herd origin. Samples were randomly chosen for 454 sequencing based on PCA scores generated from normalized Cq data obtained through the Gut Microbiotassay for 454 sequencing (see Figure 1). Circles represent K-means clustering. For further information see Additional file 2.

A Shannon’s diversity index was calculated from the grand data: control ileum = 3.48; case ileum = 3.23; control colon = 3.43; and case colon = 3.18, however, the group differences (in Shannon’s diversity indices) are negligible and their biological relevance unclear. Additionally, a PCoA was generated from the grand data that showed separation of the sequenced samples according to NNPD status (first canonical axis) and gut section (second canonical axis), as shown in Figure 4. The two major loading scores in the PCoA were explained by the following: Control: genus *Lactobacillus* and species *Fusobacterium varium*; Case: species *E. coli* and genus *Enterococcus*; Ileum: genus *Lactobacillus* and species *Fusobacterium ulcerans*; and Colon: species *F. varium* and species *Fusobacterium mortiferum* (Additional file 2)*.*

## Discussion

All piglets included in the study had previously been tested for hemolytic *E. coli*, *Cl. difficile, Cl. perfringens* type A and C, coronavirus, rotavirus species A and C, and microscopically inspected for parasites, with the conclusion that none of these agents were related to NNPD [15]*.* In this study it was generally expected that bacteria associated with diarrhoea would exhibit more or less consistent tendencies in case piglets compared with control piglets. Consequently, the Cq results and the sequencing results displaying similar trends will be discussed in relation to diarrhoea in the subsequent section, with the main focus being on potential NNPD-causing bacteria with high statistical estimates (Tables 2, 3 and 4). Sequencing results are based on a small subset of case and control piglets. The mapped sequences are mainly referred to as most similar species or isolate identified, while acknowledging the limits of classifying bacteria at these taxonomic levels. Nonetheless, even though mapped sequencing results at species and particularly isolate level are questionable, these are still construed as indicative and possibly important bacteria, which is why they are highlighted in the consecutive text.

### Genus *Enterococcus*

Enterococci were 24.15 times more abundant in diarrhoeic piglets than in healthy piglets (*p* <0.0001), and moreover genus *Enterococcus* was one of the bacterial groups positively related with status, meaning that the presence of Enterococci increases the risk of suffering from NNPD (odds ratio 1.40 per ln unit of *Enterococcus, p* = 0.009). Genus *Enterococcus* was classified to 27 different OTUs, of which *Enterococcus hirae*, *Enterococcus faecium*, *Enterococcus durans*, and *Enterococcus mundtii* were the predominant.

Enterococci are ubiquitous distributed in nature and a consistent finding in the gastrointestinal tract of several animal species, including the pig [13,18,27,28]. An association of Enterococci to neonatal porcine diarrhoea has been reported in previous studies [13,18,29,30]. Another noteworthy feature is that this genus is reported to be resistance to several antibiotics [27,28], which could explain why the efficiency of antibiotic treatment of NNPD is variable.

Larsson *et al.* found neonatal porcine diarrhoea (NPD) in Swedish pig farms to be associated with enteroadherent *E. hirae* colonizing the small intestine accompanied by mucosal lesions [29]. This conforms to observations published by Jonach *et al.* [18] using FISH to investigate the same piglets as the present study. The authors demonstrated small intestinal colonization by adherent *Enterococcus* spp. and often found them occurring together with adherent *E. coli* [18]. According to sequencing results, *E. hirae* was the most abundant species, consistent with results from a former study on Enterococcal communities in pig feces [27]. Nonetheless, this species displayed the smallest difference in numbers of OTU between diarrhoeic and healthy piglets’ gut microbiota. However, the numerical occurrence of reads may not be related to the development of diarrhoea, especially not if diarrhoea is a result of co-occurrence of different bacteria such as *E. hirae* and *E. coli*.

*E. durans* could be a contributor to NNPD, as it has previously been reported to co-occur with *E. coli* in cases of neonatal piglet diarrhoea [13,23]. In a study from 1984 in which foals and gnotobiotic piglets were experimentally inoculated with *E. durans*, this species was found to promote the proliferation of *E. coli*, and to adhere to the mucosa of the small intestine in either case [24]. In fact, *E. durans* has been hypothesized to act as a primary enteric pathogen with the ability to clear the way for other pathogens [23]. Although the ratio of *E. durans* between case and control gut sections is largest for colonic content, there were 40% more reads of this species in the ileum compared with the colon, in line with previous findings [13,23].

The preceding information implies an interesting co-occurrence of Enterococci and *E. coli* [18]. Similarly, this study also found a significant effect of the interaction between species *E. coli* and genus *Enterococcus,* which contributed to the risk of piglets suffering from NNPD (*p =* 0.02). Furthermore, there were generally more Enterococci in the ileum compared with the colon, which supports previous findings (*p =* 0.02) [13,18,23,29]. Both *E. hirae* and *E. durans* have been reported to cause villous atrophy, which was frequently found in the small intestine of piglets included in this study [18,23,29].

*E. mundtii* was almost exclusively found in the gut microbiota of case piglets, primarily in antibiotic-treated piglets, indicating that antibiotic treatment created a favorable environment for this species. Interestingly, this finding did not account for one of the antibiotic-treated animals, in which no *E. mundtii* was detected.

### Phylum Fusobacteria

Piglets with diarrhoea harbored more bacteria classified to phylum Fusobacterium (*p =* 0.02). A similar trend has been associated with acute hemorrhagic diarrhoea in dogs and ulcerative colitis in humans [31,32]. In this study, 16 OTUs were identified. Species *F. mortiferum* was a consistent finding in the digesta of all piglets, but it was less abundant in control piglets compared to case piglets. Nonetheless, Portrait *et al.* demonstrated that this species was able to produce bacteriocin-like substance(s) with an inhibitory effect on a number of both Gram-positive and Gram-negative species [33]. Therefore, it is difficult to say whether the increased abundance of *F. mortiferum* in case piglets is due to its potential pathogenicity or if the increase is a type of defense mechanism mediated by the gut microbiota. Phylum Fusobacterium was one of the two bacterial groups that were significantly reduced by antibiotic treatment (*p =* 0.002).

### Class Beta- and Gammaproteobacteria, family Enterobacteriaceae and species *Escherichia coli*

*E. coli* are commonly found in the gastrointestinal tract. This species is normally a harmless commensal in the host, nonetheless some *E. coli* possess pathogenic features [34,35].

This study found a highly significant effect of the interaction between NNPD status and this bacterial taxonomic lineage in relation to age in days (Figure 2 and Table 3). Thus, with increasing age, these bacteria proliferated in piglets with diarrhoea (though not significantly), whereas members of this group were significantly reduced in control piglets. With age, the estimated difference increased, indicating that the diarrhoeic piglets have impaired ability to control and clear this group of bacteria. However, it also suggests that NNPD is associated with an imbalanced gut microbiota, creating a favorable environment for these bacteria.

Kongsted *et al.* [15] thoroughly examined the same animals for *E. coli* by aerobic culturing, serogrouping, and testing for virulence factor genes. The most prevalent finding was the presence of non-hemolytic *E. coli,* independent of diarrhoeic status. Fimbrial genes were evenly distributed among the genes investigated in approximately 25% of the tested *E. coli* from both case and control piglets. Of all piglets, only one case of classic Enterotoxigenic *E*. c*oli* and six cases of non-typable hemolytic *E. coli* were detected, all in diarrhoeic piglets [15].

NNPD piglets investigated by 454 sequencing from different pig farms resulted in the identification of 109 OTUs and revealed a remarkably large number of reads classified to DEC8A and *E. coli* HQ219945.1.1457, both with the highest prevalence in case animals in all but one pig. The single pig that stood out was from pig farm four, which generally differed from the others. DEC8A is the name of a diarrhoeagenic *E. coli* (DEC) isolate classified as EHEC 2 (enterohemorrhagic *E. coli* 2 clonal complex), serotype O111a:NM [36]. EHEC 2 comprises a group of pathogenic *E. coli* reported to cause various disorders in mammals, including diarrhoea. The O111a was not part of the serogroups investigated by Kongsted *et al.* [15].

Except for one nucleotide positioned in the 5′-end of the *E. coli* oligonucleotide probe used for FISH analysis by Jonach *et al.* [18], this probe matches the DEC8A 23S rRNA gene [18,37]. Hence, the possibility cannot be dismissed that the *E. coli* adherent to the small intestinal epithelium observed by Jonach *et al.* [18] could be DEC8A. This could also explain the frequent finding of villous atrophy previously found to characterize diarrhoeic piglets [15,18].

*E. coli* HQ219945.1.1457 isolate was detected in ileal and colonic content of all animals sequenced, though in much higher numbers in diarrhoeic piglets, indicating that it might contribute to the pathogenesis of NNPD.

Piglets born to gilts had a significantly higher abundance of family Enterobacteriaceae and species *E. coli* than piglets born to multiparous sows (*p =* 0.03, 0.004, respectively). These were mainly classified to be the aforementioned isolates: DEC8A and *E. coli* HQ219945.1.1457. The increased risk of having NNPD if born to a gilt is consistent with farmers’ and veterinarians’ anamnesis reports, as they describe NNPD to be most prevalent among litters from first parity sows [2,13,14,17]. While the frequency of piglets born to gilts in the data material was 38% as a product of the randomized selection of sows, and the frequency of case piglets born to gilts was 66%, the odds ratio of NNPD for being born to a gilt from the logistic regression model (2) was estimated at 25.28 (*p* < 0.0001), reflecting the strongly significant effect of being born to a gilt (see also Table 4). The logistic regression model also found that piglets of different NNPD status could be separated based on colonic colonization with species *E. coli* from class Gammaproteobacteria according to 454 sequencing results.

### Phylum Firmicutes, class Bacilli, genus *Lactobacillus,* and genus *Streptococcus*

These bacterial groups are all part of the normal gut microbiota of pre-weaned piglets [4,38]. The gut microbiota of case piglets was inhabited by fewer *Lactobacillus acidophilus, Streptococcus gallolyticus subsp. gallolyticus, Streptococcus alactolyticus,* and *Streptococcus uberis* compared with the gut microbiota of control piglets.

Lactobacilli have been shown to colonize the intestines of piglets soon after birth and to be a stable member of the gut microbiota throughout the intestinal tract [4]. A low abundance of species *L. acidophilus,* which is regarded as a beneficial bacterium, could be an indicator of a troubled gastrointestinal milieu in pigs, as it was also diminished in diarrhoeic piglets included in this study. *S. gallolyticus subsp. gallolyticus* has previously been isolated from the gastrointestinal tract of numerous animal species, including pigs, and is therefore most likely included in the normal gut microbiota [39,40]. Additionally, *S. alactolyticus* is a member of the normal gut microbiota in pigs. There are few reports of *S. uberis* being isolated from pigs [41,42], and it does not seem to play a significant role in the development of NNPD, as the abundance of *S. uberis* is significantly lower in case piglets versus control piglets.

### Phylum Firmicutes, family Clostridium cluster I, species *Clostridium perfringens*, family Clostridium cluster IV, and family Clostridium cluster XIV

In general, there were fewer of these bacteria in intestinal content from both the ileum and colon of case piglets compared with control piglets, except that colonic content possessed a higher number of *Cl. perfringens* (not statistically significant). It can be speculated whether the increased abundance in the colon reflects the reduction of Cl. cluster I and *Cl. perfringens* in the ileum (*p* = 0.01, 0.04, respectively). Nonetheless, 454 sequencing of amplicons generated by primer sets targeting Cl. cluster I and higher taxonomical levels failed to demonstrate this trend. *Cl. perfringens* is a normal finding in the gastrointestinal tract but it is also a potential pathogen [43]. Sequencing amplicons generated by the primer set targeting family Cl. cluster I revealed reads from 17 OTUs, of which only *Cl. perfringens* ATCC 13124 was worthy of notice. *Cl. perfringens* ATCC 13124 is a type A strain that is a potential diarrhoea-causing agent [43]. However, because piglets included in this study have all been tested for *Cl. perfringens* type A (among others) previously [15], where it was a frequent finding with higher prevalence in control piglets versus case piglets, this species is not considered to be essential to the development of NNPD.

The fact that all remaining bacterial groups were diminished in diarrhoeic piglets is most likely due to an imbalance in the gut microbiota because all of these groups have been demonstrated in digesta from healthy piglets [38]. Various bacteria from family Cl. cluster IV and Cl. cluster XIV are regarded to be beneficial due to their ability to produce short chain fatty acids (SCFAs) such as acetate, propionate, and butyrate [44].

Of the 64 OTUs classified to family Cl. cluster IV, deficient bacteria mainly comprised species *Ruminococcus bromii*, which have previously been found in the porcine gastrointestinal tract [40]. A reduced number of *R. bromii* have also been reported in patients suffering from ulcerative colitis [32].

Family Cl. cluster XIV was not significantly related to the NNPD status of the pigs, but there was a significant effect of suffering from diarrhoea for a prolonged period of life (percentage of diarrhoea) that resulted in a reduction in the abundance of this family (*p* = 0.001). Thus, this bacterial group was indirectly affected by NNPD, as results indicate that family Cl. cluster XIV was vulnerable to the continuously flushing effect of diarrhoea. A scarce population of bacteria from family Cl. cluster XIV has also been described in previous studies on gut microbial communities in intestinal disorders [31,45]. *Roseburia inulinivorans* was the central bacterium missing from the 81 OTUs classified to family Cl. cluster XIV. This species is able to degrade oligofructose to free fructose, which can function as a fuel for other members of the gut microbiota, a phenomenon called cross-feeding [46].

### Phylum Bacteroidetes and genus *Bacteroides*

Members of phylum Bacteroidetes are a common finding in the gastrointestinal tract of mammals, and they are also an early intestinal colonizer of the healthy piglet [4,5,38,40]. Culture studies found bacteria belonging to this phylum in piglets older than 48 hours, most frequently in the large intestine [4]. This result is consistent with the findings of the present study, which found phylum Bacteroidetes to be more abundant in colonic contents of both case piglets and control piglets. However, diarrhoea resulted in a significant depletion of these bacteria in the colon (phylum Bacteroidetes *p* = 0.0009, and genus *Bacteroides p* = 0.04). Several studies have also found a reduced presence of these bacteria in different enteric disorders, such as acute non-hemorrhagic diarrhoea in dogs, experimentally induced swine dysentery in pigs, and inflammatory bowel disease in humans [31,45,47]. A total of 126 OTUs were identified. Unfortunately, sequencing results did not reveal any consistent tendencies for this taxonomic lineage. Disregarding the criterion that piglets from all herds should exhibit the same trends, case piglets harbored particularly lower numbers of species *Bacteroides pyogenes, Bacteroides rodentium, Bacteroides xylanisolvens*, and the unclassified *Porphyromonadaceae bacterium* C941 in their intestinal luminal content versus control piglets.

### Phylum Actinobacteria

Several studies have found phylum Actinobacteria to be part of the normal gut microbiota and to be scarce in the gut microbiota of different gastrointestinal disorders, such as irritable bowel syndrome in humans and acute hemorrhagic diarrhoea in dogs [31,48]. The phylum includes the genus *Bifidobacterium*, which is considered to be beneficial to its host, and a number of species from this genus are recognized probiotics [49,50]. There was a significant depletion of members from this phylum in the intestinal content of NNPD-affected piglets compared with those of control piglets (*p* < 0.0001). This was supported by OTU counts from all diarrhoeic piglets, except for the piglet that originated from the atypical herd mentioned earlier. Of the 74 OTUs the species scarcely represented were *Bifidobacterium boum*, and *Corynebacterium kutscheri* and they were mainly diminished from the colon. *Bifidobacterium* as probiotic has been demonstrated to protect piglets against weaning diarrhoea associated with *E. coli*, and *B. boum* to inhibit Shiga toxigenic *E. coli* virulence gene expression experimentally [51,52]. This fits the fact that case piglets harbored an inverse proportion of members from the taxonomical lineage class Beta- and Gammaproteobacteria, family Enterobacteriaceae and species *E. coli,* and from phylum Actinobacteria, having a lot more of the former bacteria and fewer of the latter.

*C. kutscheri* has primarily been reported to be a common bacterium on the mucous membranes of rodents including the gastrointestinal tract [53]. Interestingly, *C. kutscheri* was more plentiful in the intestinal content of healthy piglets than in that of diarrhoeic piglets, irrespective of gut section. The bacterium does not count as part of the normal porcine gut microbiota in the existing literature. Thus, even though this cannot be ruled out for certain, it is possible that piglets may have acquired this species from foraging rodents directly or indirectly.

### Class Deltaproteobacteria

This bacterial group was not associated with the diarrhoeic status of the piglets, but there was a significant reduction in numbers of members of this class in case piglets suffering from prolonged diarrhoea (increasing percentage of lifetime). Moreover, Deltaproteobacteria were also diminished by administration of antibiotics to the piglets. These facts are intertwined, as case piglets were mainly given antibiotics in an attempt to cure prolonged diarrhoea. Finally, both results were not strongly significant (*p* = 0.04, *p* = 0.02, respectively). The only OTU found by sequencing amplicons generated by the primer targeting this class was species *Desulfovibrio piger,* and this was only identified in healthy piglets from two herds*.* This species has primarily been described as an opportunistic pathogen in humans but has also been detected in the feces of wild ducks [54,55]. While the species was primarily found in colonic content of the control piglets, it is postulated that this species constitutes part of the normal gut microbiota of piglets.

### PCoA of the gut microbiota

From the BION-meta processed sequencing data a PCoA was generated (Figure 4). This investigation supported the preliminary PCA that was produced from the Gut Microbiotassay data, and additionally the major loading scores confirm the findings in the section above concerning the different bacterial groups. It diverges from the preceding discussion by taking all bacteria into account, and the clustering strongly suggests that it is possible to associate certain bacteria with a healthy gut microbiota.

### The intestinal microbiota of the aging piglet

When the data were subset according to age in days, this study found that, with age, the gut microbiota clustered in relation to status, which probably reflects the significance of *E. coli* in NNPD (Figure 3). Control piglets had significantly fewer of this species compared with case piglets, and over time the abundance of *E. coli* diminished in control piglets whilst it increased in case piglets (Figure 2). A more defined microbiota with increasing age is in line with conclusions from a previous study [56], which found that seven- to nine-day-old piglets (youngest piglets included in the study) had the lowest individual average similarity, independent of environmental or maternal relationship, compared with piglets from older age groups. The more distinctive clustering of NNPD status with increasing age could hint age to be a relevant factor in the diagnosis of NNPD.

### Antibiotics

The NNPD piglets investigated in this study included antibiotic-treated piglets. Antibiotic-treated piglets were included because they typically suffered the worst cases of NNPD and were medicated for ethical reasons. It is evident that antibiotics have an impact on the gut microbial composition [6]. However, one of the characteristics of NNPD is that it responds poorly to antibiotics. Additionally, Figures 1, 3 and 4 illustrate how it is possible to cluster piglets according to NNPD status despite the fact that some of the NNPD cases are treated with different antibiotics, indicating that the effect of NNPD on the gut microbial distribution and composition is more significant than the effect exerted by antibiotics in piglets of this age group.

### Considerations on study approach

Case and control piglets included in this study all originate from herds affected with NNPD, hence the study lack the inclusion of true control piglets from non-affected herds. Nonetheless, at the time of selecting the herds it was virtually impossible to find a true control herd due to the unknown aetiology and the much limited knowledge of NNPD. This also means that despite great efforts to ensure correct inclusion of NNPD herds through predefined criteria there is still a risk that piglets actually did not suffer from NNPD.

It is acknowledged that this study only examines the bacterial composition of the luminal content, and not bacteria from the gastrointestinal wall. However, bacteria associated with the mucosa are not restricted to this site, as they are shed into the lumen together with intestinal epithelial cells and mucus. Thus, it is assumed that this study also detects these or at least a sub-fraction of these.

Additionally, results from this study are affected by the various steps involved in preparing the samples, for example DNA extraction, PCR, and sequencing, which all introduce variation into the final outcome [57]. Furthermore, the results do not reflect bacterial numbers but relative values of 16S and 23S rRNA gene abundance or OTUs (consensus sequences). Conclusions drawn from sequencing data are based on OTUs displaying similar trends to data obtained from the Gut Microbiotassay. Moreover, sequences had to be represented in a noteworthy number, and with somewhat consistent findings in piglets, irrespective of the variables in relation to diarrhoea. This means that there might be a risk that important bacteria could be overlooked if they do not meet the aforementioned criteria.

Finally, to determine causality based on the associations found in this study these should be investigated further for instance through bacterial isolation and inoculation studies.

## Conclusion

The results of this study on NNPD indicate that bacteria could be the aetiology of this diarrhoea that affects piglets during the first week of life. At present, it is impossible to conclude whether diarrhoea is a consequence of an absence of beneficial bacteria or if diarrhoea is an outcome of invading pathogenic bacteria or an overgrowth of opportunistic pathogenic bacteria. *E. coli* appears to be a contributing factor to NNPD as NNPD piglets differ from healthy piglets by colonic colonization of bacteria from class Beta- and Gammaproteobacteria, primarily being species *E. coli*, and it appears that there is an important effect of age, both of which might be relevant in the characterization and diagnosis of NNPD. There is reason to believe that genus *Enterococcus* is a participating factor, most likely species *E. hirae* or species *E. durans.* Furthermore, diarrhoeic piglets appear to suffer from an imbalanced gut microbiota, in which bacteria regarded as beneficial are diminished, which particularly accounts for *L. acidophilus*. The associations found in this study are currently being investigated further.

### Ethical

According to Danish laws no ethical approval is required for studies not including treatment groups or needle injections/blood testing. In addition no ethical approval is required for euthanization of animals by veterinarians. Therefore this study was not subject to ethical approval, but fulfilled the regulations from the Danish Ministry of Justice. Hence, all handling of animals was performed by trained personnel and veterinarians, and euthanization was executed exclusively by veterinarians. Procedures concerning the animals were all part of routine examinations and diagnosis of animals normally used in practice.

### Endnotes

^1^ Gilts: 6 and 3 weeks ante partum, Sows: 3 weeks ante partum.

^2^ ETEC: Enterotoxigenic *Escherichia coli.*

^3^ PRRS: Porcine reproductive and respiratory syndrome.

^4^ enzyme-linked immunosorbent assay.

^5^ immunoperoxidase test.

## Additional files

Additional file 1:
**Primer modifications introduced to improve their performance and accompanying Ribosomal Database Project (RDP) search results.** Nucleotide explanation: M = C/A, Y = T/C. As the legends states, this table summarizes the modifications introduced in two primer sets from the Gut Microbiotassay: “Domain Bacteria B V4-V5” and “Phylum Firmicutes”, respectively. Ribosomal Database Project search results are given as percentage coverage of intended target group to demonstrate the improved performance of the modified primers tested *in silico.*
Additional file 2:
**Loadings, eigenvalues, and explained variance by the major principal components related to Figures** **1** **,** **3** **, and** **4** **, respectively.**
Additional file 3:
**BION-meta generated output.** This file describes the BION-meta pipeline of the bioinformatics analysis of the sequencing data [NCBI:SRP044282]. In addition, it contains a link to a website holding the BION-meta output generated in this study. Finally, it also includes a link to a website from where BION-meta can be downloaded. Additional file 4:
**Results from linear discriminant analysis (LDA) of data related to Figure** **1** **and Figure** **3** **.** Linear discriminant analysis (LDA) was used to classify observations into cases or controls by their Fluidigm-derived microbial profile and to estimate how well this classification corresponded with the actual status of the sample. This classification was followed up with a multivariate analysis of variance (MANOVA) using Wilks Lambda statistic.

## Abbreviations

AA48.48: Access Array 48.48
454BL: Access Array Barcode Library for the 454 GS FLX Titanium Sequencer
Cq: Quantification cycle
DEC: Diarrhoeagenic *E. coli*
EHEC 2: Enterohemorrhagic *E. coli* 2 clonal complex
ETEC: Enterotoxigenic *E. coli*
FISH: Fluorescence in situ hybridization
ln: Natural logarithm
LDA: Linear discriminant analysis
LSU: Large subunit
NNPD: New neonatal porcine diarrhoea
NPD: Neonatal porcine diarrhoea
OTU: Operational taxonomic units
PCA: Principal component analysis
PCoA: Principal coordinates analysis
PRRS: Porcine reproductive and respiratory syndrome
qPCR: Quantitative real-time polymerase chain reaction
rRNA: Ribosomal RNA
SCFA: Short chain fatty acids
SSU: Small subunit
