# Supplementary material for: Characterization of the bacterial gut microbiota of piglets suffering from new neonatal porcine diarrhoea
Source: BMC Vet Res. 2015 Jun 23;11:139. doi: 10.1186/s12917-015-0419-4 (PMC4476181; doi:10.1186/s12917-015-0419-4)
Supplement: Additional file 1: — Primer modifications introduced to improve their performance and accompanying Ribosomal Database Project (RDP) search results. Nucleotide explanation: M = C/A, Y = T/C. As the legends states, this table summarizes the modifications introduced in two primer sets from the Gut Microbiotassay: “Domain Bacteria B V4-V5” and “Phylum Firmicutes”, respectively. Ribosomal Database Project search results are given as percentage coverage of intended target group to demonstrate the improved performance of the modified primers tested in silico. [file 12917_2015_419_MOESM1_ESM.docx]

**Additional file 1: Primer modifications introduced to improve their performance and accompanying** **Ribosomal Database Project (RDP) search results.** Nucleotide explanation: M = C/A, Y = T/C.

| Primer | Sequence (5′→ 3′) | RDP search^1^, % coverage | |
| --- | --- | --- | --- |
|  |  | **Domain Bacteria** | **Phylum Firmicutes** |
| Domain Bacteria B V4-V5 *(r)* | CCGTCAATTCCTTTGAGTTT [68] | 51.3 | - |
|  | CCGTCAATTCMTTTGAGTTT | 58.9 | - |
| Phylum Firmicutes *(f)* | CTGATGGAGCAACGCCGCGT [69] | - | 6 |
|  | CTGAYGGAGCAACGCCGCGT | - | 37.4 |

^1^: RDP searches were performed using default settings with no mismatches allowed.

**References:**

68. Schwieger F, Tebbe CC: **A new approach to utilize PCR-single-strand-conformation polymorphism for 16s rRNA gene-based microbial community analysis.** *Appl Environ Microbiol* 1998, **64:**4870-4876.

69. Haakensen M, Dobson CM, Deneer H, Ziola B: **Real-time PCR detection of bacteria belonging to the Firmicutes Phylum.** *Int J Food Microbiol* 2008, **125:**236-241.
