# Supplementary material for: Characterization of the bacterial gut microbiota of piglets suffering from new neonatal porcine diarrhoea
Source: BMC Vet Res. 2015 Jun 23;11:139. doi: 10.1186/s12917-015-0419-4 (PMC4476181; doi:10.1186/s12917-015-0419-4)
Supplement: Additional file 3: — BION-meta generated output. This file describes the BION-meta pipeline of the bioinformatics analysis of the sequencing data [NCBI:SRP044282]. In addition, it contains a link to a website holding the BION-meta output generated in this study. Finally, it also includes a link to a website from where BION-meta can be downloaded. [file 12917_2015_419_MOESM3_ESM.docx]

**Additional file 3: BION-meta generated output.** The workflow recipe, parameters, inputs and outputs can be easily navigated via the static web site: [http://104.131.104.167/~mlhh/.](http://104.131.104.167/~mlhh/)

Silva profile tables summarize the mapped sequences. The MID numbers corresponds to the following:

| MID | Status | Age | Gut section | Herd |
| --- | --- | --- | --- | --- |
| 18 | case | 5 | ileum | 1 |
| 19 | case | 5 | colon | 1 |
| 23 | control | 5 | ileum | 1 |
| 25 | control | 5 | colon | 1 |
| 26 | case | 6 | ileum | 3 |
| 27 | case | 6 | colon | 3 |
| 28 | control | 6 | ileum | 3 |
| 30 | control | 6 | colon | 3 |
| 31 | case | 5 | ileum | 2 |
| 32 | case | 5 | colon | 2 |
| 33 | control | 5 | ileum | 2 |
| 34 | control | 5 | colon | 2 |
| 35 | case | 5 | ileum | 4 |
| 36 | case | 5 | colon | 4 |
| 37 | control | 5 | ileum | 4 |
| 38 | control | 5 | colon | 4 |

Briefly, the workflow steps were as follows: 1) separation by sample barcodes and primers; 2) removal of primer remnants and low quality sequence at the ends, as well as filtering by length (200 nucleotides) and overall quality (96%); 3) removal of chimeric sequences. The following steps were performed for the forward and reverse phylogenetic primer separately, as well as in combination: 4) separation of each sample by matching with the phylogenetic primer(s); 5) matching all sequences against the SSU and LSU Silva datasets and producing a table with the highest 1% similarities for each query; 6) mapping the similarities to the Silva SSU and LSU taxonomies by summing up the original read numbers to create scores for each taxon; and 7) formatting these scores and producing tables that show them. The end result is a table for each combination of sample primers and phylogenetic primers.

The as yet unpublished open source package BION-meta can be downloaded from <http://box.com/bion> (Larsen N *et al.*, in prep.).
