# Supplementary material for: Characterization of the bacterial gut microbiota of piglets suffering from new neonatal porcine diarrhoea
Source: BMC Vet Res. 2015 Jun 23;11:139. doi: 10.1186/s12917-015-0419-4 (PMC4476181; doi:10.1186/s12917-015-0419-4)
Supplement: Additional file 4: — Results from linear discriminant analysis (LDA) of data related to Figure 1 and Figure 3 . Linear discriminant analysis (LDA) was used to classify observations into cases or controls by their Fluidigm-derived microbial profile and to estimate how well this classification corresponded with the actual status of the sample. This classification was followed up with a multivariate analysis of variance (MANOVA) using Wilks Lambda statistic. [file 12917_2015_419_MOESM4_ESM.docx]

# Linear discriminant analysis (LDA)

Linear discriminant analysis (LDA) based on leave-one-out cross-validation was used to classify observations into Case/Control by their Fluidigm-derived microbial profile and to estimate how well this classification corresponded with the actual status of the sample. This classification was followed up with a multivariate analysis of variance (MANOVA) using Wilks Lambda statistic. It was not possible to use LDA on the sequencing data as a consequence of the small number of samples (16 samples against 231 OTUs).

# Results:

## Figure 1:

|  |  | classification | |  |  |
| --- | --- | --- | --- | --- | --- |
|  |  | Case | Control |  | Correct |
| Status | Case | 70 | 29 |  | 0.707071 |
|  | Control | 18 | 90 |  | 0.833333 |

MANOVA *p* for classification by Status = 2.507e-15

## Figure 3:

### Day 3

|  | Case | Control |  | correct |
| --- | --- | --- | --- | --- |
| Case | 20 | 10 |  | 0.66667 |
| Control | 8 | 23 |  | 0.74194 |

*p =* 0.003628

### Day 4

|  | Case | Control |  | correct |
| --- | --- | --- | --- | --- |
| Case | 12 | 5 |  | 0.70588 |
| Control | 8 | 12 |  | 0.6 |

*p* = 0.03665

### Day 5

|  | Case | Control |  | correct |
| --- | --- | --- | --- | --- |
| Case | 27 | 9 |  | 0.75 |
| Control | 5 | 32 |  | 0.86486 |

*p* = 2.70E-05

### Day 6-7

|  | Case | Control |  | correct |
| --- | --- | --- | --- | --- |
| Case | 14 | 2 |  | 0.875 |
| Control | 0 | 20 |  | 1 |

*p* = 1.16E-05
